# Application of discrete wavelet transform for analysis of genomic sequences of *Mycobacterium tuberculosis*

**DOI:** 10.1186/s40064-016-1668-9

**Published:** 2016-01-22

**Authors:** Shiwani Saini, Lillie Dewan

**Affiliations:** Department of Electrical Engineering, National Institute of Technology, Kurukshetra, Haryana 136119 India

**Keywords:** Discrete wavelet transform, *Mycobacterium tuberculosis*, Genomic sequences, Signal analysis

## Abstract

This paper highlights the potential of discrete wavelet transforms in the analysis and comparison of genomic sequences of *Mycobacterium tuberculosis* (MTB) with different resistance characteristics. Graphical representations of wavelet coefficients and statistical estimates of their parameters have been used to determine the extent of similarity between different sequences of MTB without the use of conventional methods such as Basic Local Alignment Search Tool. Based on the calculation of the energy of wavelet decomposition coefficients of complete genomic sequences, their broad classification of the type of resistance can be done. All the given genomic sequences can be grouped into two broad categories wherein the drug resistant and drug susceptible sequences form one group while the multidrug resistant and extensive drug resistant sequences form the other group. This method of segregation of the sequences is faster than conventional laboratory methods which require 3–4 weeks of culture of sputum samples. Thus the proposed method can be used as a tool to enhance clinical diagnostic investigations in near real-time.

## Background

Human tuberculosis (TB) is caused by an intracellular pathogen, *Mycobacterium tuberculosis* and it replicates rapidly in the lungs with high oxygen concentration. The genome of MTB is approximately 4.4 million base pairs long and is one of the largest known bacterial genomes. According to WHO statistics (2015), in the year 2014 an estimated 9.6 million people developed TB and 1.5 million died from the disease. Global TB control measures are affected by the emergence of drug resistant, multidrug resistant and extensively drug resistant strains. Resistance in these MTB strains to anti-TB drugs occurs due to chromosomal mutations. Out of the 480,000 cases of multidrug-resistant TB (MDR-TB) estimated to have occurred in 2014, only about a quarter of these were detected and reported.

Tuberculosis disease control can be achieved by determining drug resistance, which is a major challenge. There are several diagnostic tests for TB that include sputum smear analysis, mycobacterium culture and X-rays. Culture-based drug susceptibility testing (DST) is considered the most significant determinant of drug susceptibility as it can define resistance irrespective of the molecular mechanism responsible for resistance. Testing of antibiotic resistance to anti-TB drug is done by isolation and culture of the bacteria followed by exposure to antibiotic drug. This method takes 3–4 weeks and also requires extensive biosafety facilities. During this time patients may not receive appropriate treatment, and drug resistance may become amplified. Moreover high burden countries lack adequate laboratory facilities. Genotyping methods have also been developed that differentiate between bacterial strains by examining specific target regions associated with drug resistance. Main diagnostic tests available commercially are the Xpert MTB/RIF assay (Cepheid, Inc.) (USFDA 2013), INNO-LiPA TB test (Innogenetics) (Morgan et al. 2005) and the GenoType MTBDRplus kit (Hain Lifescience) (Ling et al. 2008). These assays have been approved by the World Health Organization as a tool for rapid MDR-TB diagnosis (WHO 2008). Genotypic tools are faster and are hence better in terms of diagnostic usefulness but require detailed information about the mutations that cause drug resistance. This is due to their inability to detect resistance due to mutations outside target regions or because they may detect inactive or incomplete resistance genes in a specimen, which are not associated with resistance to the antimicrobial drug under test (Fournier et al. 2013).

Whole genome sequencing (WGS) has the potential to overcome such problems. WGS is a promising multi-purpose genotyping tool, which can be used both for prediction of drug susceptibility as well as epidemiological investigations. Though aspects of cost-efficiency and the appropriate setting for the implementation of WGS techniques are not yet well established but with the current ongoing research and development, bacterial genomes can now be sequenced in a few hours with the help of bench top analyzers (Brown et al. 2015) and at reduced costs due to high throughput (Gardya 2015). WGS methods can not only analyze known mutation sites associated with resistance but can also help analyze other loci indicating the presence or absence of resistance. This can help health care professionals to analyze the entire genome in terms of disease related variants (Wlodarska et al. 2015). Thus whole genome sequencing is capable of extending rapid testing to the full range of antibiotics, which can expedite the access to the required line of treatment and hence minimize the exposure of patient to ineffective drugs. Several methods based on WGS of MTB sequences such as conception of new prophylactic and therapeutic interventions (Cole et al. 1998), factors influencing its transmission (Guerra-Assunção et al. 2015), identification of outbreak-related transmission chains (Roetzer et al. 2013), prediction of drug susceptibility and resistance (Walker et al. 2015) have been reported in literature.

Apart from molecular methods based on whole genome sequences of MTB, signal processing of complete genomic sequences can help display and explore structural patterns capable of being interpreted and compared. Graphical representations obtained from signal processing methods can provide insight into the evolution, structure and function of genomes (Anastassiou 2000). With the huge amount of genomic data available after the completion of genome sequencing projects, rapid analysis of genomic data is possible using signal processing methods. These methods help characterize DNA sequences by distinct visual patterns using graphical representations in comparison to conventional laboratory methods (Cristea et al. 2007; Nandy et al. 2006). Several graphical approaches for genomic sequence analysis such as DNA walks (Berger et al. 2003), Z-curves (Zhang et al. 2003), Fourier transforms, phase analysis (Cristea 2003) and wavelet transforms (Lorenzo-Ginori et al. 2009) have been reported in literature. DNA walk has been used as a tool to visualise changes in nucleotide composition, locating coding and non coding regions, identifying periodicities and large scale local and global features present in many genomes (Li’O 2003; Haimovich et al. 2006). Fourier transforms have been used to determine periodicities in proteins, identification of protein coding DNA regions and open reading frames (Zhou et al. 2007). Z-curves have been used in identifying replication origins of archaeal genomes (Zhang and Zhang 2005). Phase analysis has been used to report the existence of global helicoidal wrapping of DNA sequences (Cristea 2003), determining pathogen drug resistance in HIV, H5N1 (Cristea 2006).

Continuous wavelet transforms have been used as an effective tool to localize events, such as the active sites prediction in protein sequences of HIV, Haemoglobin Human α protein (Rao and Swamy 2008), fractal analysis of DNA sequences (Voss 1992). Discrete wavelet transforms have been used to identify gene locations in genomic sequences (Ning et al. 2003), determining focal genomic aberrations in single nucleotide polymorphism (Hur and Lee 2011), determining pattern irregularities (Haimovich 2006), predict the ori and ter regions of bacterial chromosomes (Song et al. 2003), identifying long-range correlations, determining base change locations (Saini and Dewan 2014), locating periodicities in DNA sequences (Vannucci and Liò 2001), detecting change points in genomic copy number data (Yu et al. 2010), analysis of G + C patterns (Dodin et al. 2000), analysing the information content in human DNA (Machado et al. 2011), analysing sequence contexts in indels of DNA sequences (Kvikstad et al. 2009).

Of all the graphical methods, wavelet transforms have the advantage of time–frequency analysis of signals. They also have the advantage of analysing signals at different frequency resolutions or scales (called multiresolution analysis) and hence are capable of determining the hidden variations in patterns of complete genomic sequences at various scales. Decomposition of a signal at a coarse scale can be used to view the trend of the whole sequence while decompositions at fine scales are used to determine single base patterns for local features. These multi resolution wavelet decompositions of complete genomic sequences can be used to investigate the similarity of various sequences at different resolution levels without the pre-requisite of sequence alignment and consideration of insertion, deletion events unlike the conventional method-BLAST. Correlation measures between different sequences at various scales of decomposition can help investigate the extent of similarity. Lower values of correlation relate to lesser sequence similarity whereas higher values of correlation are significant of higher structural similarity. This can help characterize scale wise disparities for each sequence as well as compare different sequences of DNA. Basic Local Alignment Search Tool (BLAST) is the most common method to ascertain sequence similarity which works by first aligning a query sequence with a subject sequence. The results are reported in the form of a ranked list followed by a series of individual sequence alignments and various statistics and scores. However for very large sequences with length of the order of million base pairs, the alignments and similarity scores are shown for different sub-sequence segments of varying lengths and not for the whole contiguous sequence. Hence the overall similarity of the complete sequence cannot be evaluated at one go.

In this paper the potential of discrete wavelet transform for comparison of MTB sequences with different resistance characteristics has been investigated. DWT has been employed to analyse and compare different strains of MTB sequences at various decomposition levels by graphical and statistical measures. Comparison of the plots of GC content of all MTB sequences has also been carried out.

### Wavelet transforms

A waveform of finite duration and zero average value is called a wavelet. WT is calculated using a mother wavelet function ψ(t), by convolving the original signal f(t) with the scaled and shifted version of the mother wavelet described by Eq. 1 where a is called the scaling parameter and b is called the translational parameter.

Mathematical transforms such Fourier Transforms (FT) and Short Time Fourier Transform (STFT) are also used in signal processing and analysis. Whereas FT only gives information about the various frequency components in a particular signal, STFT provides the time–frequency localization of the signal but in a fixed window frame. Wavelet transforms in comparison to FT and STFT, offer the advantage of time frequency localisation of a signal by using windows of varying sizes and hence are capable of multi resolution of signals. There are two types of wavelet transforms: continuous wavelet transforms (CWT) and discrete wavelet transforms (DWT).

1 $$Cab = \int_{t} {f\left( t \right)\frac{1}{\sqrt a }\frac{{\psi *\left( {t - b} \right)}}{a}{\text{d}}{\text{t}}}$$

**Fig. 1:**
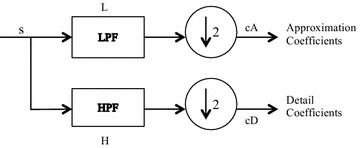
Signal decomposition

**Fig. 2:**
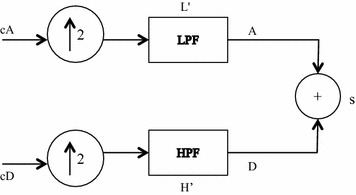
Signal reconstruction

**Fig. 3:**
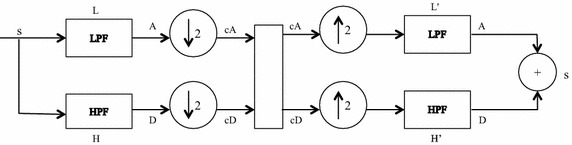
Signal decomposition and reconstruction

Since continuous wavelet transforms are calculated at all possible scales and positions, they generate a large amount of data and require larger computation time. In discrete wavelet analysis, scales and positions are chosen based on powers of two called the dyadic scales. After discretization the wavelet function is defined as given in Eq. 2:

2 $$\uppsi_{\text{m,n}} \left( {\text{t}} \right) = \frac{1}{{\surd 2^{\text{m}} }}\uppsi^{*} \frac{{\left( {{\text{t}} - {\text{nb}}_{0 } {\text{a}}_{0}^{{{\text{m}} }} } \right)}}{{{\text{a}}_{0}^{\text{m}} }}$$

where a_0_ and b_0_ are constants. The scaling term is represented as a power of a_0_ and the translation term is a factor of a_0_^m^. Values of the parameters a_0_ and b_0_ are chosen as 2 and 1 respectively and is called as dyadic grid scaling. The dyadic grid wavelet is expressed in Eq. 3 as

3 $$\psi_{m,n} \left( t \right) = \frac{1}{{\sqrt {2^{m} } }}\psi \frac{{\left( {t - n2^{m } } \right)}}{{2^{m} }} = 2^{{ - \frac{m}{2}}} \psi \left( {2^{ - m} t - n} \right)$$

where *ψ*_*m*,*n*_(*t*) represents the wavelet coefficients at scale m and location n. This dyadic scaling scheme is implemented using filters developed by Mallat (2000). The basic filtering process is represented in Fig. 1. The original signal is filtered through a pair of high pass filter g(n) and low pass filter h(n) and then down sampled to get the decomposed signal through each filter which is half the length of the original signal. This process of filtering results in decomposition of the signal into different frequency components. The low frequency components are called approximations and high frequency components are called details. This constitutes one level of decomposition, mathematically expressed as

4 $$Y_{hp} \left( k \right) = \mathop \sum \limits_{n} X\left( n \right)g\left( {2k - n} \right)$$

5 $$Y_{lp} \left( k \right) = \mathop \sum \limits_{n} X\left( n \right)h\left( {2k - n} \right)$$

where X(n) is the original signal, h[n] and g[n] are the sample sequences or impulse responses and *Yhp*(*k*) and *Ylp*(*k*) are the outputs of the high-pass and low-pass filters, respectively, after subsampling by 2. This procedure, known as sub-band coding, can be repeated for further decomposition. At every level, the filtering and subsampling results in half the number of samples (and hence half the time resolution) and half the frequency band spanned (and hence double the frequency resolution). The signal S after one level of decomposition can be expressed as S = cD + cA (Fig. 1). After the decomposition, the original signal can be synthesized using inverse discrete wavelet transform. The signal is reconstructed as shown in Fig. 2 by up sampling of the decomposed signal followed by filtering through two complementary filters (L′ and H′) and is expressed as A + D = S. The low-pass and high-pass decomposition filters (L and H) and reconstruction filters (L′ and H′) together form a set of quadrature mirror filters as shown in Fig. 3.

**Fig. 4:**
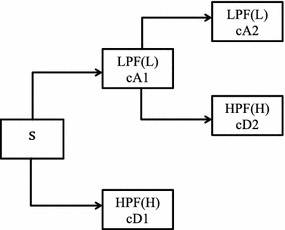
Multilevel decomposition of signal

**Fig. 5:**
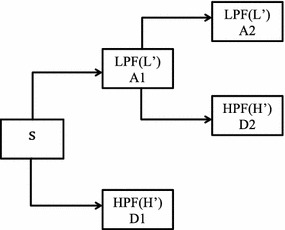
Multilevel reconstruction of signal

The resolution of the signal is a measure of the amount of detail information in the signal, can be changed by the filtering operations, and the scale can be changed by up sampling and down sampling operations. The decomposed signal can be broken down into lower resolution components by decomposing the successive approximations iteratively. Signal decomposition at different frequency bands is successive high-pass and low-pass filtering and forms the basis of multi resolution decomposition (Fig. 4). The signal can be analyzed at different frequency bands and resolutions by decomposing the signal into a coarse approximations and details. Similar relationships also hold for the reconstructed signal (Fig. 5). The decomposed signal can be written as *s* = *cA*2 + *cD*2 + *cD*1. Similarly the signal can be reconstructed from the successive approximations and details as *A*2 + *D*2 + *D*1 = *s*.

With the decomposition of the original signal into components of different scales, DWT provides a powerful tool to detect the patterns of variations across scales in observed data. The following statistical parameters of the wavelet decompositions can be calculated and compared between different sequences.

1. Energy of a signal x(n) decomposed into approximations a_n_ and details d_n_ at a particular scale m is given as
  6 $$\mathop \sum \limits_{n = 1}^{N} \left| {x\left( n \right)^{2} } \right| = \mathop \sum \limits_{n = 1}^{N} \left| {a_{n}^{m} } \right|^{2} + \mathop \sum \limits_{m = 1}^{M} \mathop \sum \limits_{n = 1}^{N} \left| {d_{n}^{m} } \right|^{2}$$
2. Wavelet variance, which is a scale-by-scale decomposition of variance of signal. It is calculated at a particular scale m as
  7 $$\left\langle {T^{2}_{m,n} } \right\rangle_{m} = \mathop \sum \limits_{n = 0}^{{2^{{\left( {M - m} \right)_{ - 1} }} }} \frac{{(T_{m,n} )^{2} }}{{2^{M - m} }}^{{}}$$
  where *T*_*m*,*n*_ represents the discrete wavelet coefficients and 2^*M*^ (=*N*) is the total number of data points in a signal. Wavelet variance is a measure of the average energy per coefficient at each scale.
3. Fluctuation intensity (FI) measures the energy distribution across different scales of decomposition. It is calculated as
  8 $$FI = \frac{{\left[ {\left\langle {T^{4}_{m,n} } \right\rangle_{m} - \left( {\left\langle {T^{2}_{m,n} } \right\rangle_{m} } \right)^{2} } \right]^{1/2} }}{{\left\langle {T^{2}_{m,n} } \right\rangle_{m} }}$$
  Fluctuation intensity is also called coefficient of variation and measures standard deviation in the variance of coefficient energies at scale m.
4. Correlation is a measure of the strength of linear relationship between variables. The correlation coefficient r_xy_ of two random variables X and Y with expected values μ_x_ and μ_y_ and standard deviation σ_x_ and σ_y_ is given by
  9 $$r_{xy} = \frac{{Cov\left( {X,Y} \right)}}{{\upsigma{\text{x}} \cdot\upsigma{\text{y}}}}$$
  where Cov(X,Y) is the covariance function between two variables X and Y. Correlation values lie between +1 and −1. Whereas the values of *r*_*xy*_ close to 1 suggest linear relationship between X and Y, values close to −1 suggest anti-correlation between the two variables and values close to 0 suggest no relationship between the two variables. Correlation coefficients can be used to evaluate the measure of similarity between different sequences.

### DNA

DNA is the main nucleic genetic material of the cells. There are four kinds of nitrogenous bases found in DNA that constitute the genomic sequences: thymine (T) and cytosine (C)—called pyrimidines, adenine (A) and guanine (G)—called purines. Nucleotide A always pairs with T while nucleotide C always pairs with G. Hence, the two strands of a DNA helix are complementary and contain exactly the same number of A, T nucleotides and the same number of C, G nucleotides. In order to apply graphical representation techniques, DNA sequences need to be mapped into their corresponding numerical values for visualization and analysis with digital signal processing methods. In this paper, DNA walk method (Berger et al. 2002) is used for mathematical representation wherein, pyrimidines (nucleotides C, T) are assigned a value of +1 and purines (nucleotides A, G) are assigned a value of −1. A DNA walk is then calculated for a particular DNA sequence as given by Eq. 10.

10 $$Y\left( i \right) = \mathop \sum \limits_{n = 1}^{N} x\left( n \right)$$

where *x*(*n*) is the numerical value of the nucleotide base in a given DNA sequence. The DNA sequences can also be represented in the form of GC (Guanine–Cytosine) content. GC content is an important parameter of bacterial genomes which has been used to scan the basic makeup of the genome, as well as to understand its coding sequence evolution. A genome shows marked variations in its GC content within a long region of its sequence in contrast to the background GC content for the whole genome. GC-rich regions include many protein coding genes, and thus determination of GC ratio helps in identifying gene-rich regions of the genome. G + C content for the whole sequence is calculated as ratio of sum of G, C bases to the sum of A, G, C, T bases (Eq. 11).

11 $$GC\;content = \frac{nG + nC}{nA + nG + nC + nT}$$

where nA, nG, nC, nT represent the number of A, G, C, T nucleotide bases respectively in a sequence. The GC content can also be calculated for a part of the sequence using sliding window technique where GC content is calculated for fixed length of a sequence called window.

## Results

**Fig. 6:**
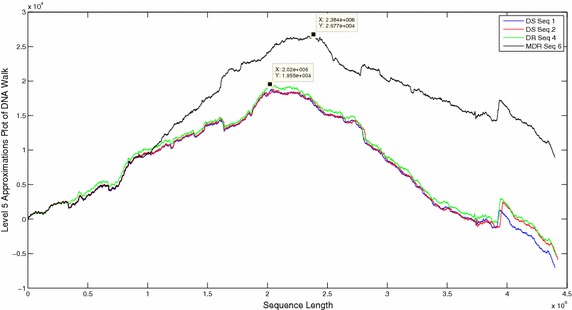
Level 5 approximation plots of DNA walk

**Fig. 7:**
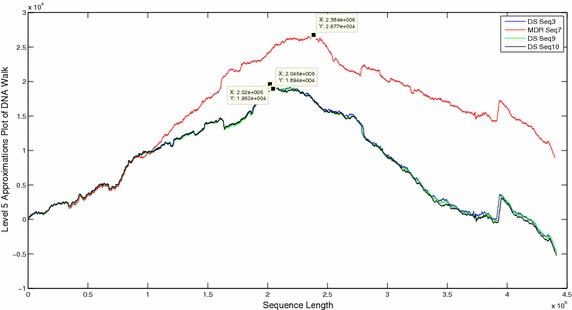
Level 5 approximation plots of DNA walk

**Fig. 8:**
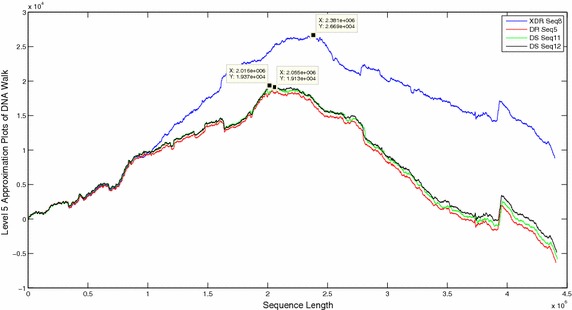
Level 5 approximation plots of DNA walk

The DNA walks of all sequences were decomposed and approximation coefficients were compared at level 5 (Figs. 6, 7, 8). Visual comparison of patterns in the approximation coefficients of DR and DS sequences showed almost similar plots in close proximity but the MDR and XDR sequences showed significantly higher peaks. The scalograms of all the sequences were also compared. Since 99 % energy of the entire sequence was contained only in the approximation coefficients, the statistical parameters of only level 5 approximations of all the sequences were compared (Table 1). The energy contained in approximation coefficients of MDR and XDR sequences is much higher than that of DS and DR sequences. Wavelet variance of the MDR and XDR sequences was also higher in magnitude in comparison to the DS and DR sequences. Fluctuation Intensity is a statistical measure of the dispersion of data points in a data series around the mean. Comparison of FI values showed that the XDR and MDR sequences exhibited values less than 1 whereas all DS and DR sequences showed FI values of greater than 1.

**Table 1:** Statistical estimates of MTB sequences

Sequence number	NCBI accession number	Resistance type	Energy (×10^14^)	Variance (×10^7^)	Fluctuation intensity	Mean (×10^3^)	Average GC content
Seq1	CP002992	DS	4.5134	4.5311	1.0651	7.5697	0.6560
Seq2	NC_009565	DS	4.5574	4.36	1.0717	7.072	0.6561
Seq3	CP001641	DS	4.875	4.1582	1.0814	8.3212	0.6561
Seq4	CP001642	DR	4.8752	4.3167	1.0552	8.2148	0.6559
Seq5	CP001664	DR	4.4055	4.3616	1.0770	7.5048	0.6563
Seq6	NC_012943	MDR	12.885	5.5967	0.7220	15.395	0.6561
Seq7	CP001658.1	MDR	12.855	5.5967	0.7220	15.395	0.6561
Seq8	NC_018078	XDR	12.866	5.6036	0.7243	15.377	0.6561
Seq9	NC_021251	DS	4.794	4.1725	1.0663	8.1778	0.6561
Seq10	NC_000962	DS	4.7932	4.284	1.0587	8.1134	0.6561
Seq11	NC_009525	DS	4.7418	4.261	1.0637	7.9384	0.6561
Seq12	CP002884	DS	4.794	4.172	1.0633	8.1778	0.6561

To quantify the similarity in the structural organization of these sequences, correlation measures were evaluated for the level 5 approximation coefficients (Table 2). From the values of correlation coefficients, it is evident that all DS, DR sequences are very similar to each other as they possess correlation values of around 0.99. The two MDR sequences and one XDR sequence are also highly correlated to each other as observed from their correlation coefficients nearing 1. At the same time the correlation values of 0.65–0.69 between the DS/DR and XDR/MDR sequences suggest that the DS and DR sequences possess different structural and sequence organization in comparison to the XDR and MDR sequences. Correlation value of 1 for the two MDR sequences (seq6, 7) and two DS sequences (seq10, 12) shows that these sequences exhibit perfect similarity in nucleotide content.

**Table 2:** Correlation coefficients

	Seq1	Seq2	Seq3	Seq4	Seq5	Seq6	Seq7	Seq8	Seq9	Seq10	Seq11	Seq12
Seq1	1	0.9952	0.9963	0.9972	0.9980	0.6526	0.6526	0.6551	0.9948	0.9963	0.9959	0.9948
Seq2		1	0.9961	0.9973	0.9976	0.6743	0.6743	0.6765	0.9978	0.9963	0.9993	0.9978
Seq3			1	0.9987	0.9969	0.6975	0.6975	0.6997	0.9986	0.9984	0.9969	0.9986
Seq4				1	0.9988	0.6754	0.6754	0.6766	0.9986	0.9990	0.9980	0.9986
Seq5					1	0.6558	0.6558	0.6611	0.9974	0.9974	0.9985	0.9980
Seq6						1	1	0.9988	0.6984	0.6876	0.6802	0.6984
Seq7							1	0.9988	0.6984	0.6876	0.6802	0.6984
Seq8								1	0.7005	0.6897	0.6823	0.7005
Seq9									1	0.9994	0.9986	1
Seq10										1	0.9989	0.9994
Seq11											1	0.9986
Seq12												1

**Fig. 9:**
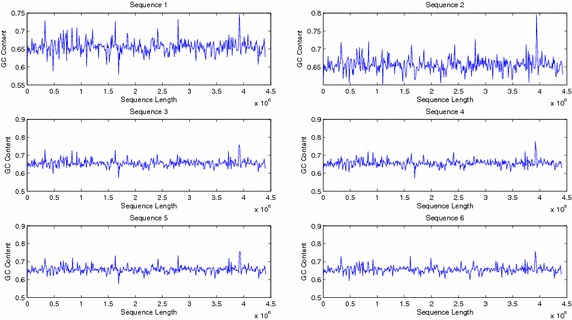
Windowed GC content for sequences 1–6

**Fig. 10:**
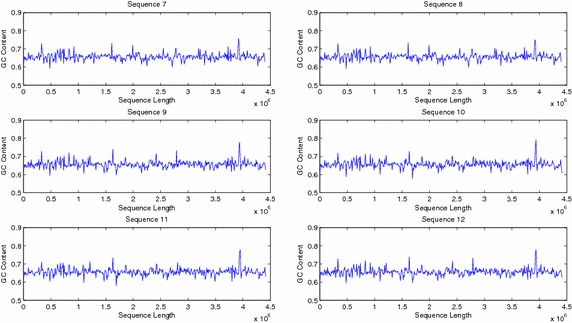
Windowed GC content for sequences 7–12

**Fig. 11:**
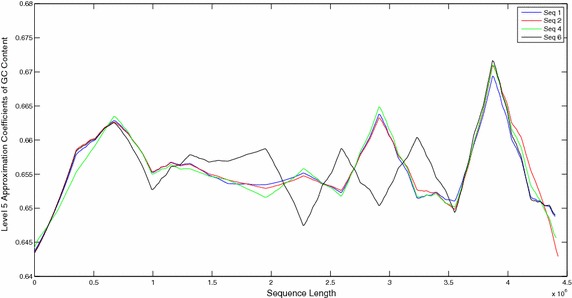
Level 5 approximation plots of GC content

**Fig. 12:**
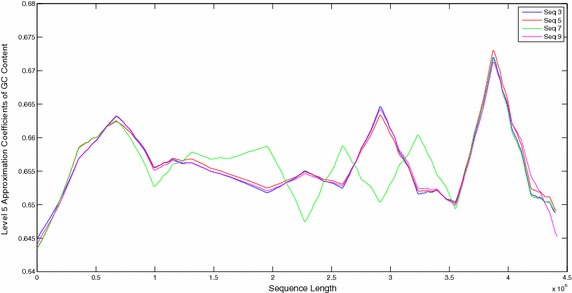
Level 5 approximation plots of GC content

**Fig. 13:**
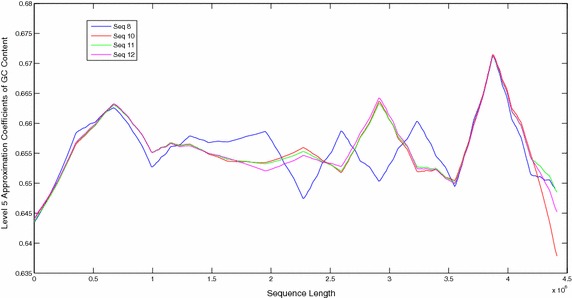
Level 5 approximation plots of GC content

The sequences were also compared by plotting their windowed GC content (Figs. 9, 10). Plots of windowed GC content cannot compare the sequences for similarities/dissimilarities except for locating the maxima and minima of GC content for a particular sequence. However wavelet plots of level 5 approximations of windowed GC content show peaks in specific regions along the complete sequences which can be compared visually (Figs. 11, 12, 13). The locations of the peaks can help in identifying gene rich regions. From the Figs. 11, 12, 13, it is observed that the locations of positive and negative peaks of all the drug susceptible and drug resistant sequences are overlapping with only slight deviations in their peak values. This suggests that in these sequences the genes are located at identical locations with only slight differences in the magnitude of GC content. However, MDR and XDR sequences showed significantly different plots. In the region between 1 Mbase to 3.5 Mbases along the sequence, most of their peaks appeared shifted with the positive peaks exhibiting significantly lower values and a negative peak of a much higher value in comparison to the peaks in plots of DS and DR sequences. Thus the organisation of the GC content of the XDR and MDR sequences is significantly different from that of DS and DR sequences. This suggests that the gene rich regions in MDR and XDR sequences are not located at similar locations as in DS and DR regions.

Thus from all the results it is observed that the wavelet coefficients of MDR and XDR sequences possess similar statistical estimates but their parameters are totally different in magnitude when compared with the DR and DS sequences. Of all the estimates, energy is the most distinguishing parameter. The energy of MDR and XDR sequences is nearly three times the energy of DR and DS sequences. Therefore it can be used to segregate the sequences broadly into two groups- one group which contains the DR and DS MTB while the other group contains the XDR and MDR MTB. Any unknown sequence can be categorised as DS or DR if it possesses energy magnitude roughly around 5 × 10^14^ while if the energy of the sequence is more than 10 × 10^14^, the sequence can be categorised as XDR or MDR.

## Conclusions

Several features of genomic sequences of MTB, irrespective of their length can be visualized using DWT analysis. The plots of multiresolution decompositions of the sequences can be used to interpret the regions of biological interest underlying them. Such multi resolution decompositions are not possible with other signal processing techniques. Apart from the visual representations, statistical approaches such as correlation using DWT can facilitate the determination of similarity between different sequences with lengths of the order of millions of bases without the need of sequence alignment and insertion–deletion events to be considered in comparison to BLAST. Therefore wavelet transforms can provide a faster method of assessing and interpreting sequences based on their nucleotide content. DWT decomposition plots can also help identify the patterns underlying the GC content that can be visualised to identify gene rich regions. The control of drug resistant TB relies on preventing the amplification of drug resistance as well as timely diagnosis of drug-resistant disease. This DWT based method can help identify the broad category of the resistance type from the complete sequence and thus can be used as an additional method along with conventional sequence based methods for development of new diagnostic tools.

## Methods

Different MTB sequences (Ilina et al. 2013): DR, MDR, XDR and DS were downloaded from NCBI (National Center for Biotechnology Information 2012) database for comparison (Table 1). To apply the signal processing techniques, the DNA sequences were mapped into a mathematical representation. DNA walks of all the mathematically represented sequences were then analyzed using discrete Haar wavelet transform. The sequences were decomposed up to 5 levels of decomposition. Statistical measures of energy, wavelet variance, fluctuation intensity, and correlation for each of the decomposed sequences were evaluated and compared. The GC content of all the sequences was also evaluated and plotted using a sliding window of 10,000 bases. The GC plots were then analyzed using DWT. The pattern differences of different sequences were visualized by comparing their approximation coefficients plots.
